# Artificial intelligence-based prognostic model accurately predicts the survival of patients with diffuse large B-cell lymphomas: analysis of a large cohort in China

**DOI:** 10.1186/s12885-024-12337-z

**Published:** 2024-05-22

**Authors:** Huilin Peng, Mengmeng Su, Xiang Guo, Liang Shi, Tao Lei, Haifeng Yu, Jieyu Xu, Xiaohua Pan, Xi Chen

**Affiliations:** 1 Department of Lymphatic Oncology, Zhejiang Cancer Hospital, Hangzhou Institute of Medicine (HIM), Chinese Academy of Sciences, Hangzhou, Zhejiang 310022 China; 2 Zhejiang University of Science & Technology, Hangzhou, Zhejiang 310027 China; 3 Department of Pharmacy, Zhejiang Cancer Hospital, Hangzhou Institute of Medicine (HIM), Chinese Academy of Sciences, Hangzhou, Zhejiang 310022 China; 4 Binjiang Institute of Zhejiang University, Hangzhou, Zhejiang 310053 China

**Keywords:** Diffuse large B-cell lymphoma, Heterogeneous, Prognostic model, Molecular era

## Abstract

**Background:**

Diffuse large B-cell lymphomas (DLBCLs) display high molecular heterogeneity, but the International Prognostic Index (IPI) considers only clinical indicators and has not been updated to include molecular data. Therefore, we developed a widely applicable novel scoring system with molecular indicators screened by artificial intelligence (AI) that achieves accurate prognostic stratification and promotes individualized treatments.

**Methods:**

We retrospectively enrolled a cohort of 401 patients with DLBCL from our hospital, covering the period from January 2015 to January 2019. We included 22 variables in our analysis and assigned them weights using the random survival forest method to establish a new predictive model combining bidirectional long-short term memory (Bi-LSTM) and logistic hazard techniques. We compared the predictive performance of our “molecular-contained prognostic model” (McPM) and the IPI. In addition, we developed a simplified version of the McPM (sMcPM) to enhance its practical applicability in clinical settings. We also demonstrated the improved risk stratification capabilities of the sMcPM.

**Results:**

Our McPM showed superior predictive accuracy, as indicated by its high C-index and low integrated Brier score (IBS), for both overall survival (OS) and progression-free survival (PFS). The overall performance of the McPM was also better than that of the IPI based on receiver operating characteristic (ROC) curve fitting. We selected five key indicators, including extranodal involvement sites, lactate dehydrogenase (LDH), *MYC* gene status, absolute monocyte count (AMC), and platelet count (PLT) to establish the sMcPM, which is more suitable for clinical applications. The sMcPM showed similar OS results (*P* < 0.0001 for both) to the IPI and significantly better PFS stratification results (*P* < 0.0001 for sMcPM vs. *P* = 0.44 for IPI).

**Conclusions:**

Our new McPM, including both clinical and molecular variables, showed superior overall stratification performance to the IPI, rendering it more suitable for the molecular era. Moreover, our sMcPM may become a widely used and effective stratification tool to guide individual precision treatments and drive new drug development.

**Supplementary Information:**

The online version contains supplementary material available at 10.1186/s12885-024-12337-z.

## Background

Diffuse large B-cell lymphomas (DLBCLs) are the most common subtype of non-Hodgkin lymphoma (NHL), and they display clinical and biological heterogeneity. Immunochemotherapy with rituximab plus cyclophosphamide, doxorubicin, vincristine, and prednisone (RCHOP) is a widely accepted, standardized treatment for patients with newly diagnosed DLBCL [1]. Despite a high response rate, approximately 10–15% of patients are resistant to first-line immunochemotherapy [2–4], and disease relapse occurs in up to 30–40% of patients after treatment [4, 5]. Therefore, individualized treatments based on risk stratification are urgently needed.

The International Prognostic Index (IPI) is the most widely used tool for risk stratification in clinical practice; this was established before the era of immunochemotherapy [6]. The revised IPI (R-IPI) and National Comprehensive Cancer Network IPI (NCCN-IPI) are often used as prognostic models during rituximab treatments. They offer advantages in predicting prognosis after immunochemotherapy [7]. A variety of gene signatures and microenvironmental biomarkers have emerged. Tumour-associated macrophages (TAMs) are important components of the DLBCL microenvironment, and upregulation of CD163^+^ M2 TAMs has been found to be associated with inferior chemotherapy effects [8] and an unfavourable prognosis [9]. Carreras et al. also found that high-level infiltration of (CD163^+^, PTX3^+^, IL10^+^) M2c-like TAMs and low infiltration of FOXP3^+^ Tregs were associated with a poor prognosis [10]. In addition, DLBCL patients expressing PD-L1 also have a poor prognosis [11]. Therefore, several studies have attempted to incorporate molecular markers into various models [4, 7, 12–15]. Of these, the five-gene risk model (*CD163、CLEC4A、COL15A1、GABRB2、IFIT3*) reliably predicts the overall survival (OS) of DLBCL [15] patients. However, even in the current molecular era, there is no consensus among research groups on the optimal molecular technique for stratifying DLBCL patients. Thus, the models have not been widely used in routine medical practice. The IPI, R-IPI, and NCCN-IPI are still widely used to accurately predict prognosis. However, all three scoring systems share the limitation of only considering clinical indicators; molecular heterogeneity is not sufficiently taken into account [7, 16].

Gene abnormalities in *MYC*, *BCL2*, and *BCL6* are strong prognostic predictors in patients with DLBCL [17–19]. DLBCL with a *MYC* rearrangement (*MYC-R*) but not a *BCL2* rearrangement *(BCL2-R*) nor a *BCL6* rearrangement (*BCL6-R*) is termed single-hit lymphoma (SHL). DLBCL with *MYC-R* and *BCL2-R* and/or *BCL6-R* is termed double- or triple-hit lymphoma (DHL and THL, respectively). All three types were reported to be associated with a poor prognosis [20]. However, to the best of our knowledge, these markers have not been used in prognostic scoring systems.

Mathematical modelling methods with digitized clinical data have gradually been introduced to identify the most important prognostic factors of diseases and predict the incidence of events [21–24]. The use of artificial intelligence to screen for DLBCL-prognostic genes is now common [25]. One study used artificial intelligence to reveal the prognostic impact of the *MYC* and *BCL2* genes [26]. However, AI-based classification methods and models are not appropriate for routine clinical practice. In addition, few models combine clinical and genetic screening factors as prognostic indicators.

The aim of our research was to establish a new prognostic model with diverse indicators, including both clinical and molecular variables, by using an intelligent screening method. Our new model represents an advancement in both methodology and indicator selection. It is well positioned to provide improved guidance for clinical treatment, especially in the context of new drugs, and aligns with the demands of the molecular era.

## Materials and methods

### Patients and clinical features

#### Case selection

We retrospectively collected clinical information and the results of *MYC*, *BCL2*, and *BCL6* fluorescence situ hybridization (FISH) tests from 401 patients with DLBCL newly diagnosed in our hospital during the period January 2015 to January 2019. We excluded patients with incomplete clinical data and those who did not receive chemotherapy. All patients were diagnosed with DLBCL by pathologists at our hospital or after external consultations. The study was conducted in accordance with the Helsinki Declaration. All patients signed informed consent forms before inclusion.

#### Fluorescence situ hybridization

FISH experiments were performed using paraffin-embedded specimens after the pathological diagnoses. All probes and 4’,6-diamidino-2-phenylindole (DAPI) counterstains used in this study were purchased from Abbott (USA), including the MYC dual-color separation (01N63-020), BCL2 dual-color separation (05N51-020), and BCL6 dual-color separation (01N23-020) probes. According to the results of hematoxylin-eosin (HE) staining, we selected regions rich in tumor cells as hybridization regions. We counted 200 tumor cells in each specimen and assessed genetic features based on specific signal patterns. We identified gene rearrangements based on signal separation, where one fused yellow signal and two separated red and green signals were observed in > 10% of cells for the *MYC* gene, > 10% of cells for the *BCL2* gene, and > 10% of cells for the *BCL6* gene. We identified gene amplifications in the presence of more than three yellow signals (or adjacent red and green signals) in the same nucleus. Two yellow signals (or adjacent red and green signals) in the same nucleus characterized normal *MYC*, *BCL2*, and *BCL6* genes.

#### Follow-up

Patients were followed up via phone calls or the Hospital Information System. We excluded patients for whom we lacked survival outcome information. The last follow-up date was 1 January 2022. We defined OS as the time from DLBCL diagnosis to death or the last follow-up, whichever came first. Progression-free survival (PFS) was the time from DLBCL diagnosis to the first disease progression, death, or last follow-up, whichever came first.

### Modelling

#### Modelling process

**Fig. 1:**
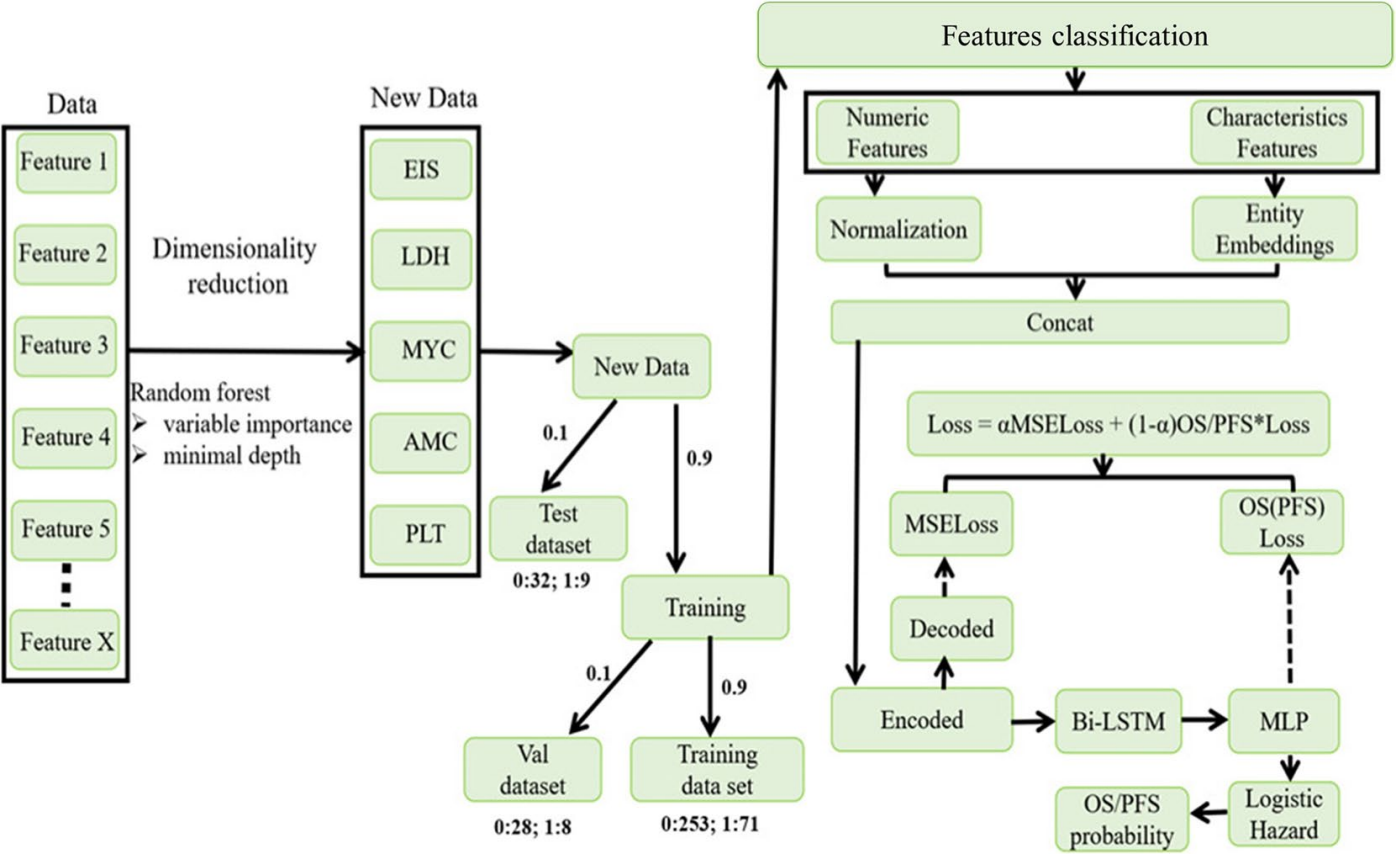
The modelling process of McPM. EIS, extranodal involvement sites; LDH, lactate dehydrogenase; AMC, absolute monocyte count; PLT, platelet count; Bi-LSTM, bidirectional long-short term memory; MLP, multi-layer perceptron; MSELoss, mean squared error loss; OS, overall survival; PFS, progression free survival

Figure 1 shows the modelling process.

#### Feature selection

We evaluated the significance of variables using the random survival forest (RSF) R package(website:https://cran.rproject.org/web/packages/randomForestSRC/index.html) [27]. Two methods were used to determine the contributions of variables to a stochastic survival model: the variable importance (VIMP) [28] method and the minimum depth method.

#### Data set segmentation

**Fig. 2:**
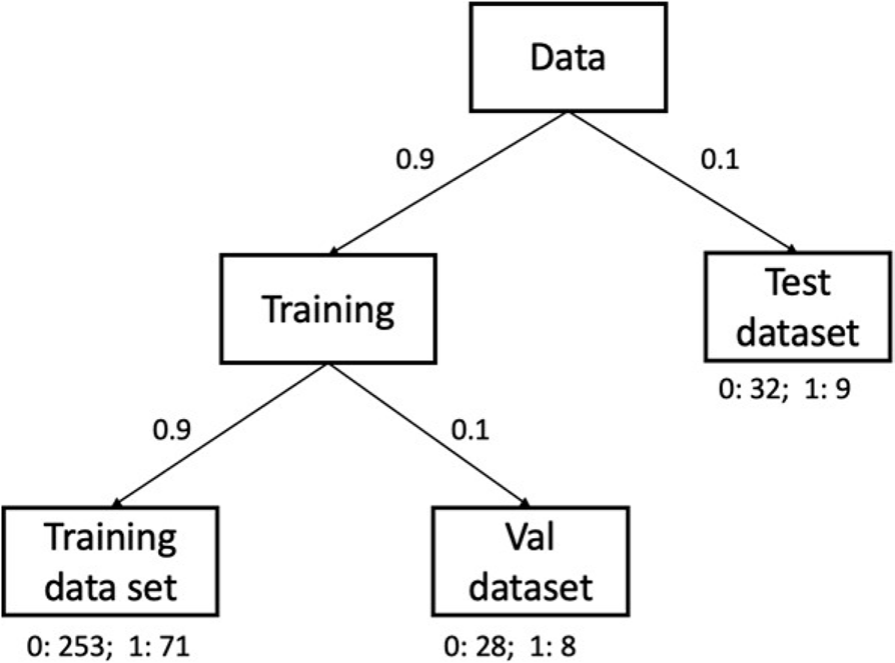
Data set segmentation. The complete data set is divided into training and test data sets in a ratio of 9:1, where the training data is further divided into training and validation data in a ratio of 9:1

We used 90% of the data for model training and 10% for model testing. We split the data used for training into two sets according to a ratio of 9:1(test data, training data, and verification data in the proportion of positive and negative samples) (Fig. 2).

#### Training

We generated a survival prediction model (“molecular-contained prognostic model”, McPM) based on entity embedding, encoded and decoded layers, bidirectional long-short term memory (Bi-LSTM), and logistic hazard techniques [29]. We classified variables into categorical, binary, and numerical categories and used them as model inputs. The encoded data were produced using the encoded layer. Future and past information was discovered and retained by the Bi-LSTM, and the final survival predictions were then generated using the logistic hazard model. The logistic hazard model is a discrete-time method and requires discretization of event times for data that are originally continuous. The model’s loss function consists of an encoder loss component (mean squared error loss, MSELoss) and a survival prediction loss component (negative logarithmic likelihood logistic regression loss, NLLLogistiHazardLoss), where the encoder loss was calculated on the basis of differences between the model’s output and input features.

#### Model evaluation

We used the concordance index (C-index) and Brier score (BS) as evaluation indexes of the survival predictions. The C-index, a commonly used indicator of survival predictions, is applied to evaluate the predictions made by an algorithm. The BS [30, 31] is used to evaluate the accuracy of a predicted survival function at a given time (t). It represents the average squared distance between the actual survival status and the predicted survival probability, and its value is always a number between 0 and 1, where 0 is the best possible value. Given a dataset of $\text{N}$ samples, $\forall \text{i} \in \left[1, \text{N}\right], (\overrightarrow{{\text{x}}_{\text{i}}},{{\updelta }}_{\text{i}},{\text{T}}_{\text{i}})$ is the format of a datapoint, and the predicted survival function is $\widehat{\text{S}}\left(\text{t}, \overrightarrow{{\text{x}}_{\text{i}}}\right), \forall \text{t} \in {\mathbb{R}}^{+}$. In the absence of right censoring, the BS can be calculated as:

$$\text{B}\text{S}\left(\text{t}\right)=\frac{1}{\text{N}}\sum\limits _{\text{i}=1}^{\text{N}}{({1}_{{\text{T}}_{\text{i}}>\text{t}}-\widehat{\text{S}}\left(\text{t}, \overrightarrow{{\text{x}}_{\text{i}}}\right))}^{2}$$

However, if the dataset contains right-censored samples, the score needs to be adjusted by weighting the squared distances using the inverse probability of censoring weights method. Let $\widehat{\text{G}}\left(\text{t}\right)=\text{P}[\text{C}>\text{t}]$ be the estimator of the conditional survival function of the censoring times calculated using the Kaplan-Meier method, where $\text{C}$ is the censoring time.

$$\text{B}\text{S}\left(\text{t}\right)=\frac{1}{\text{N}}\sum\limits_{\text{i}=1}^{\text{N}}\frac{{(0-\widehat{\text{S}}\left(\text{t}, \overrightarrow{{\text{x}}_{\text{i}}}\right))}^{2}\cdot {1}_{{\text{T}}_{\text{i}}>\text{t},{{\updelta }}_{\text{i}}=1}}{\widehat{\text{G}}\left({\text{T}}_{\text{i}}^{-}\right)}+\frac{{(1-\widehat{\text{S}}\left(\text{t}, \overrightarrow{{\text{x}}_{\text{i}}}\right))}^{2}\cdot {1}_{{\text{T}}_{\text{i}}>\text{t}}}{\widehat{\text{G}}\left(\text{t}\right)}$$

In terms of benchmarks, a useful model will have a BS below 0.25. Indeed, it is easy to see that if $\forall \text{i} \in \left[1, \text{N}\right], \widehat{\text{S}}\left(\text{t}, \overrightarrow{{\text{x}}_{\text{i}}}\right)=0.5$ then $\text{B}\text{S}\left(\text{t}\right)=0.25$.

We performed other statistical analyses using GraphPad 9.0 software. We considered *P* values < 0.05 statistically significant.

## Results

### Clinical and molecular characteristics of patients with DLBCL

Table 1 lists the clinical and molecular characteristics of the 401 patients in this study. The median age of onset was 58 years and the proportion of male participants was slightly higher than that of female participants. Nearly half of patients (49.6%) had an Ann Arbor stage of III–IV. One fifth of patients were accompanied with B symptoms. Most patients were non-germinal center B cell (Non-GCB) subtypes according to the cell of origin of lymphoma. We determined the expression of *MYC*, *BCL2*, and *BCL6* genes using FISH tests in all patients, and we found that 296 patients (73.8%) had at least one genetic abnormality. We identified 16 cases of double-hit lymphoma /triple-hit lymphoma (DHL/THL), including 1 case of THL, 10 of *MYC* and *BCL6* DHL, and 5 of *MYC* and *BCL2* DHL. In total, 381 patients received RCHOP-like regimens and only 20 (5.0%) received CHOP-like regimens. Additionally, we have also performed univariate and multivariate analyses using Log rank and COX (see Table S1 and Table S2).

**Table 1:** Clinical and molecular characteristics of the 401 patients

**Characteristics**	**All patients (*N*=** **401)**
Median age (range)	58(15–84)
Aged >60 years	170/401 (42.4%)
Male	201/401 (52.3%)
Ann Arbor III–IV	199/401 (49.6%)
ECOG ≥ 2	60/401 (14.9%)
B symptoms	85/401 (21.1%)
Extranodal sites ≥ 2	93/401 (23.2%)
ALC ≤ 1.0 × 10^9/L	114/401 (28.4%)
AMC ≥ 0.6 × 10^9/L	136/401 (33.9%)
ALC/AMC< 3: 1	197/401 (49.1%)
Albumin < 35 g/L	41/401 (10.2%)
β2 microglobulin > 3.0 mg/L	97/401 (24.2%)
LDH >240 U/L	194/401 (48.4%)
IPI	
0–2	276/401 (68.8%)
3–5	125/401 (31.2%)
Ki-67 > 70%	238/401 (59.4%)
COO	
Non-GCB	274/401 (68.3%)
GCB	127/401 (31.7%)
*MYC* gene	
Rearrangement	40/401 (10.0%)
Amplification	75/401 (18.7%)
Normal	286/401 (71.3%)
*BCL2* gene	
Rearrangement	21/401 (5.2%)
Amplification	137/401 (34.2%)
Normal	243/401 (60.6%)
*BCL6* gene	
Rearrangement	145/401 (36.2%)
Amplification	104/401 (25.9%)
Normal	152/401 (37.9%)
Double-/triple-hit	16/401 (3.9%)
RCHOP-like regimen	381/401 (95.0%)
CHOP-like regimen	20/401 (5.0%)

*Abbreviations*: *ECOG* Eastern Cooperative Oncology Group, *ALC* absolute lymphocyte count, *AMC* absolute monocyte count, *LDH* lactate dehydrogenase, *IPI* International Prognostic Index, *COO* cell of origin, *GCB* germinal-center B-cell, *RCHOP* rituximab plus cyclophosphamide, doxorubicin, vincristine, and prednisone

### Importance of variables

**Fig. 3:**
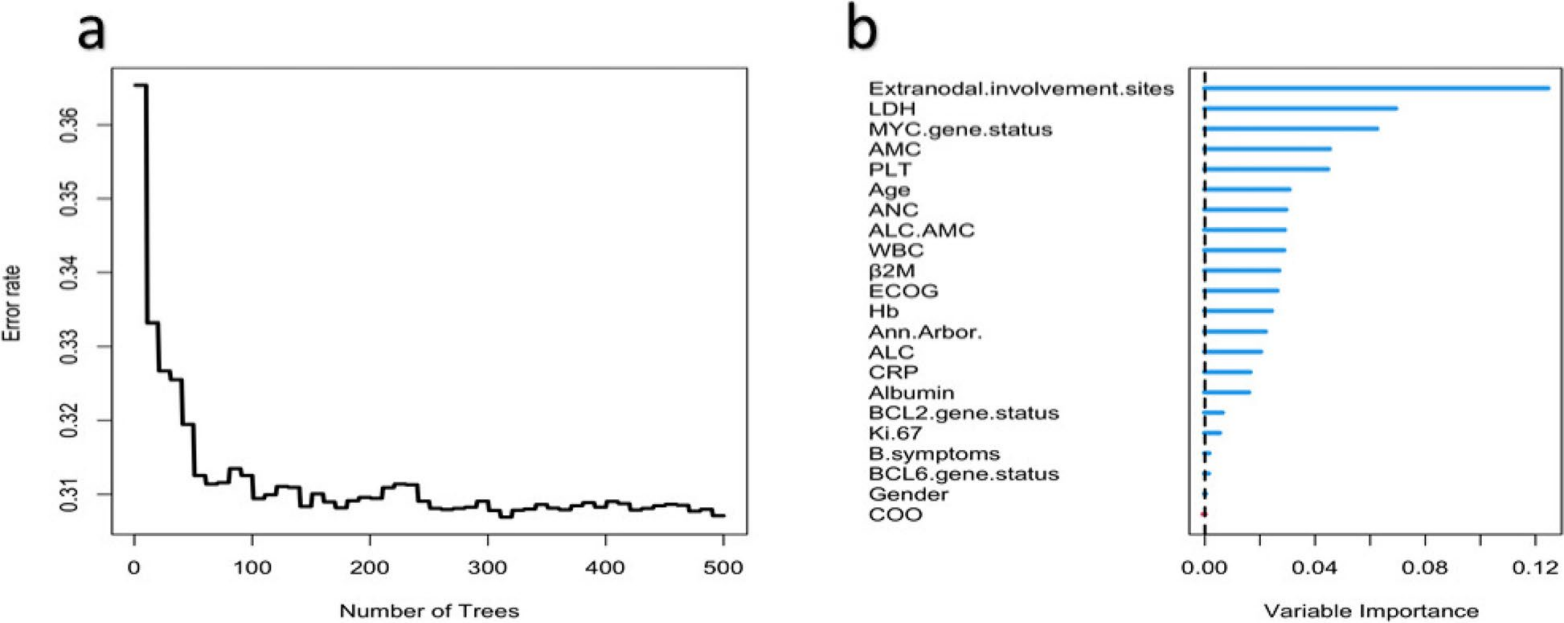
Error rate curve and feature weights. **a** Plot of error rate according to the number of survival trees; (**b**) Comparison of the importance of all 22 factors. The error rate of the model stabilizes at around 0.31. Based on the feature weights, the top five most important indicators can be identified as extranodal involvement sites, LDH, MYC gene status, AMC, and PLT. LDH, lactate dehydrogenase; ALC, absolute lymphocyte count; AMC, absolute monocyte count; ECOG, Eastern Cooperative Oncology Group score; β2M, β2 microglobulin; PLT, platelet count; ANC, absolute neutrophil count; CRP, C-reactive protein; WBC, white blood cells; Hb, haemoglobin; COO, cell of origin

We used the RSF method to assess the importance of 22 clinical and pathological variables, as shown in Fig. 3. We found that the prediction error rate decreased significantly with an increase in the number of survival trees (Fig. 3a). When survival trees increase to a certain number, the error rate curve flattens out. We found that the error rate was lowest near 300 trees (within a range of 0–500 trees).

Figure 3b lists the weights of the different variables. The top five variables ranked by weight were extranodal involvement sites, lactate dehydrogenase (LDH), *MYC* gene status, absolute monocyte count (AMC), and platelet count (PLT).

Both the VIMP and minimum depth methods are commonly used for variable screening when employing the RSF model. VIMP represents the difference between the original and the new error rate. A VIMP value < 0 indicates that the variable reduces the prediction accuracy, while a VIMP value > 0 indicates that the variable improves the prediction accuracy. The minimum depth method assesses the importance of each variable by calculating the minimum depth at which it appears in the decision tree when the tree reaches its final node.

**Fig. 4:**
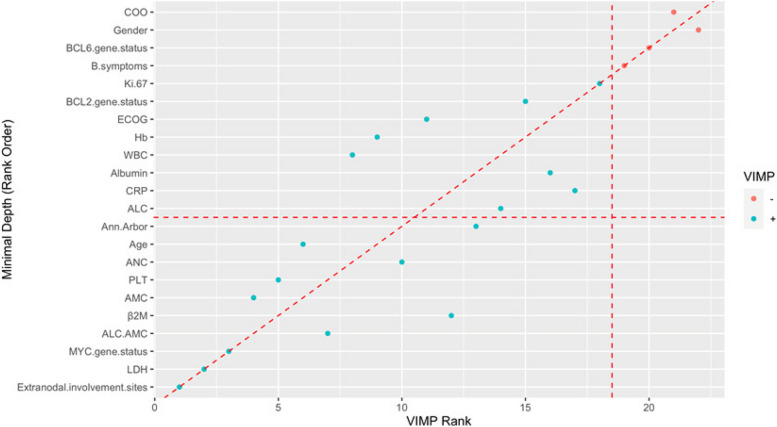
Scatter plot of the VIMP and minimum depth method. Blue points represent VIMP values > 0, and red points represent VIMP values < 0. Points on the red diagonal dotted line indicate that the same two methods rank a given variable similarly. Points above the diagonal dotted line indicate a higher VIMP ranking, and points below the diagonal dotted line indicate a higher minimum depth ranking (e.g. AMC and PLT are above the red diagonal dashed line, indicating that these two variables have a higher VIMP ranking.)LDH, lactate dehydrogenase; ALC, absolute lymphocyte count; AMC, absolute monocyte count; ECOG, Eastern Cooperative Oncology Group; β2M, β2 microglobulin; PLT, platelet count; ANC, absolute neutrophil count; CRP, c-reactive protein; WBC, white blood cells; Hb, haemoglobin; COO, cell of origin

Table 2 lists the VIMP values and depth of different variables. Figure 4 shows a scatter plot comparing the two methods. In the plot, blue points represent VIMP values > 0, and red points represent VIMP values < 0. Points on the red diagonal dotted line indicate that the same two methods rank a given variable similarly. Points above the diagonal dotted line indicate a higher VIMP ranking, and points below the diagonal dotted line indicate a higher minimum depth ranking. We obtained similar results with both variable selection methods; extranodal involvement sites, LDH, *MYC* gene status, AMC, and PLT seem to be important variables for prognosis.

**Table 2:** Importance of variables according to the minimum depth and VIMP methods

**Factors**	**Depth**	**VIMP**
Extranodal involvement sites	3.788	0.11695186
LDH	4.036	0.06482836
*MYC* gene status	4.232	0.06325718
AMC	5.07	0.04809921
PLT	5.564	0.04207818
Age	5.65	0.03592581
ALC/AMC	4.972	0.03355172
WBC	6.06	0.0332111
Hb	6.106	0.03296761
ANC	5.578	0.03135262
ECOG	6.636	0.02613268
β2M	5.054	0.02512321
Ann Arbor	5.668	0.02117884
ALC	5.752	0.0186599
*BCL2* gene status	6.674	0.01002272
Albumin	6.038	0.00930746
CRP	5.982	0.00880714
Ki-67	7.126	0.00684168
B Symptoms	7.902	-0.0002797
*BCL6* gene status	7.916	-0.0006722
COO	8.244	-0.0007618
Gender	8.006	-0.0007761

*Abbreviations*: *LDH* lactate dehydrogenase, *AMC*, absolute monocyte count, *PLT* platelet count, *ALC* absolute lymphocyte count, *WBC* white blood cells, *Hb* haemoglobin, *ANC* absolute neutrophil count, *ECOG* Eastern Cooperative Oncology Group, β*2M* β2 microglobulin, *CRP* c-reactive protein, *COO* cell of origin

### Comparison of the new McPM and the IPI for OS prediction

We assessed the goodness of fit of the two models using the C-index. A higher C-index value indicates a better model fit. The Brier score, defined as the mean square of the difference between the predicted and actual values, allowed us to calculate the integrated Brier score (IBS), an overall measure of model prediction performance. The lower the IBS, the higher the prediction accuracy of the model. Compared with the IPI, the McPM had a superior OS prediction accuracy due to its higher C-index (McPM, 0.8672 vs. IPI, 0.8025; Table 3) and lower IBS (McPM, 0.1296 vs. IPI, 0.2159; Table 3).

**Table 3:** Comparison of OS between the IPI and McPM

	**McPM**	**IPI**
C-index	0.8672	0.8025
Integrated Brier Score	0.1296	0.2159

*Abbreviations*:* IPI* International Prognostic Index, *McPM* molecular-contained prognostic model, *C-index* concordance index

**Fig. 5:**
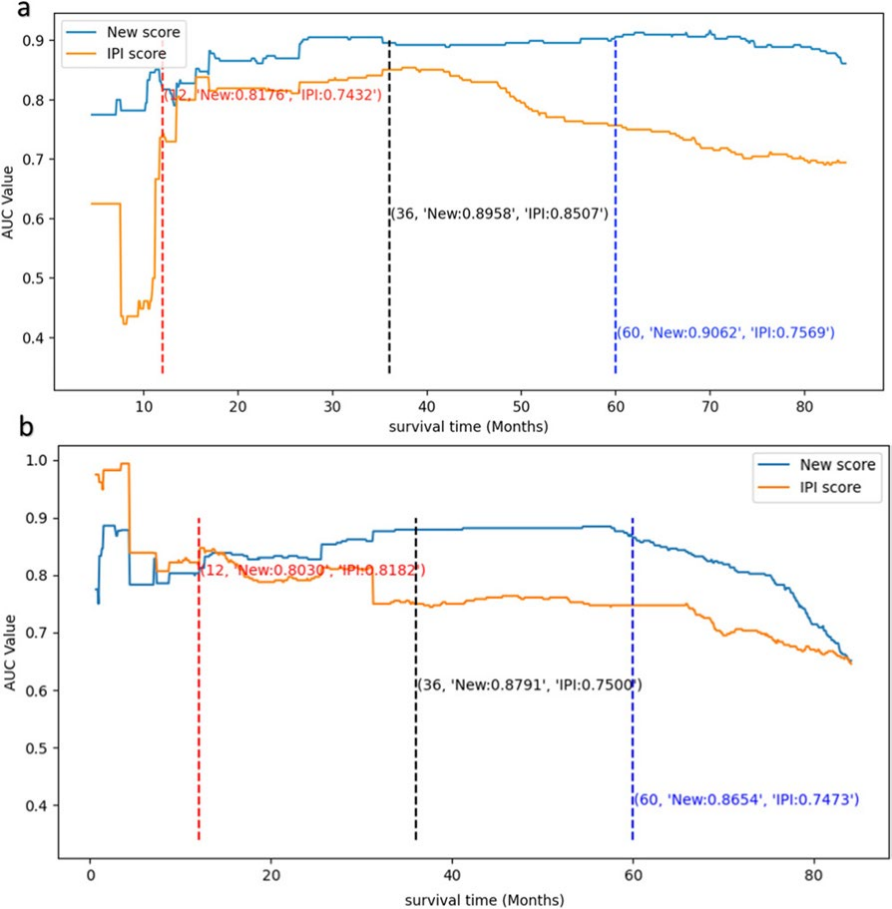
Comparison of the AUC values at various time points of the two models (McPM and IPI). **a** The relationship between survival time and the AUC value in OS prediction; (**b**) The relationship between survival time and the AUC value in PFS prediction. AUC stands for area under the curve, which is a metric used to evaluate the performance of a binary classification model. A higher AUC value suggests better discriminatory power. AUC, area under the curve; McPM, molecular-contained prognostic model; IPI, International Prognostic Index; OS, overall survival; PFS, progression-free survival

**Fig. 6:**
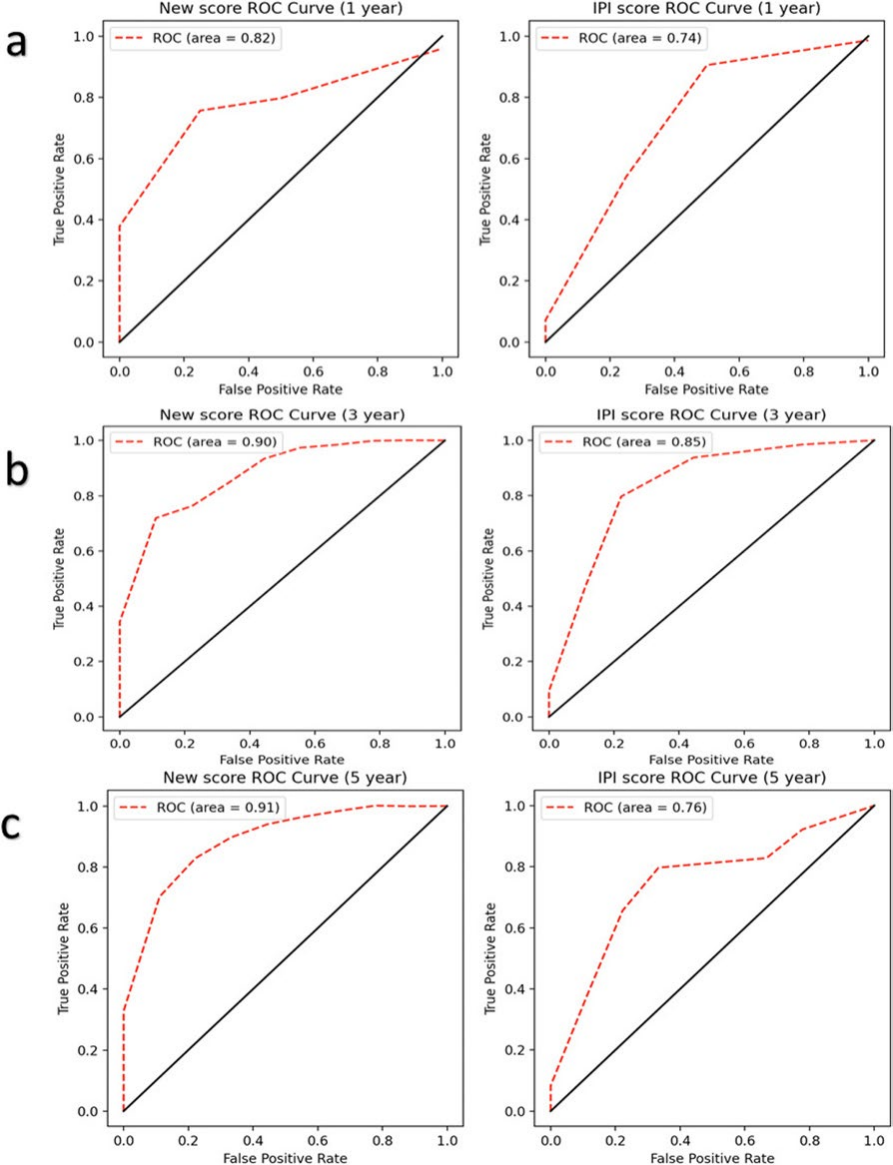
Comparison of the 1- (**a**), 3- (**b**), and 5-year (**c**) ROC curves of different models (McPM and IPI for OS prediction). It is a graphical plot that illustrates the diagnostic ability of a binary classifier system as its discrimination threshold is varied. The larger the area under the curve, the better the classification performance of the model. ROC, Receiver operating characteristic; OS, Overall survival; McPM, Molecular- contained prognostic model; IPI, International Prognostic Index

The receiver operating characteristic (ROC) curve and area under curve (AUC) are important for evaluating prognostic model discrimination. Figure 5 shows the change in AUC values for OS or PFS predictions as survival time increases. For OS prediction (Fig. 5a), the new score outperformed the IPI model over a continuous 80-month period, maintaining stable AUC values ranging from 0.8 to 0.9. In contrast, the AUC values of the IPI were less stable, with predicted AUC values ranging from 0.4 to 0.8. The predicted AUC values within 1 year ranged from 0.4 to 0.7 with large fluctuations, while the predicted AUC values after 1 year ranged from 0.7 to 0.8 with small fluctuations. The gap between the two models became wide over time. Figure 6 shows the ROC curves of the new score and the IPI models for OS predictions. Compared with the 1-, 3-, and 5-year survival AUCs of the IPI, the values of the new model increased by approximately 6%, 4%, and 15%, respectively. Overall, the new model showed a superior OS predictive performance than the IPI.

### Comparison of the new McPM and the IPI for PFS prediction

We further explored the predictive performance of the two scoring systems for PFS. Compared with IPI, The McPM provided better PFS discrimination than the IPI, as indicated by its higher C-index value (0.8394 vs. 0.7308; Table 4) and lower IBS (0.1619 vs. 0.1712; Table 4).

**Table 4:** Comparison of progression-free survival values between the IPI and the McPM

	**McPM**	**IPI**
C-index	0.8394	0.7308
Integrated Brier Score	0.1619	0.1712

*Abbreviations*: *IPI* International Prognostic Index, *McPM* molecular-contained prognostic model, *C-index * concordance index

**Fig. 7:**
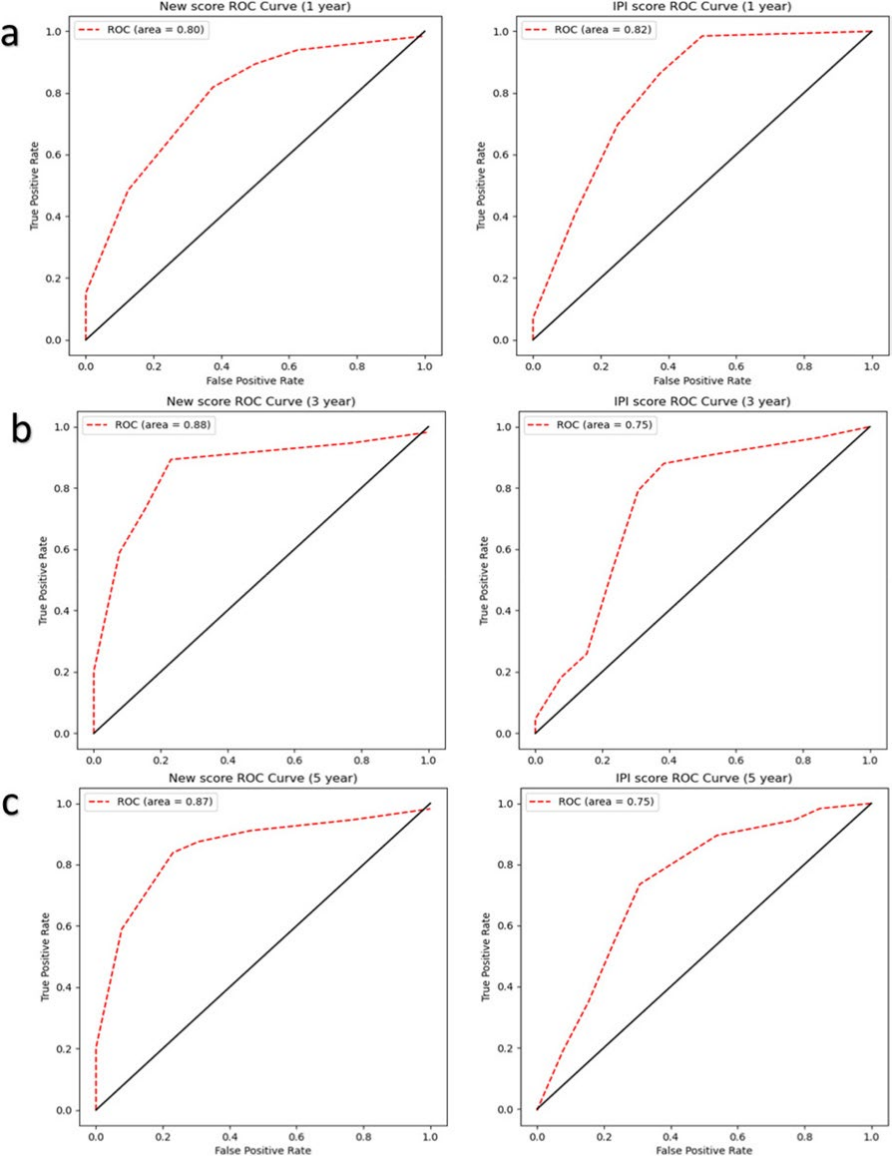
Comparison of the 1- (**a**), 3- (**b**), and 5-year (**c**) ROC curves of different models (McPM and IPI) of PFS. It is a graphical plot that illustrates the diagnostic ability of a binary classifier system as its discrimination threshold is varied. The larger the area under the curve, the better the classification performance of the model. ROC, receiver operating characteristic; PFS, progression-free survival; McPM, molecular-contained prognostic model; IPI, International Prognostic Index

The AUC curve values for PFS (Fig. 5b) also differed significantly between the two models. During the continuous 80-month period, the AUC values of the new model remained stable between 0.75 and 0.9, and they began to decrease gradually after 60 months. By contrast, the predicted IPI AUC values ranged from 0.7 to 0.8 and showed an overall decreasing trend with time. Figure 7 shows the 1-, 3‐, and 5‐year ROC curves for PFS predictions, for both the McPM and the IPI. While the 1-year AUC value for the McPM was slightly lower than that of the IPI (0.80 vs. 0.82), the new model had better AUC values for 3- and 5-year survival, by approximately 12% in both cases. Overall, despite a slightly lower 1-year AUC, the new model demonstrated better PFS predictive performance than the IPI.

### Comparison of two models with actual outcomes

**Fig. 8:**
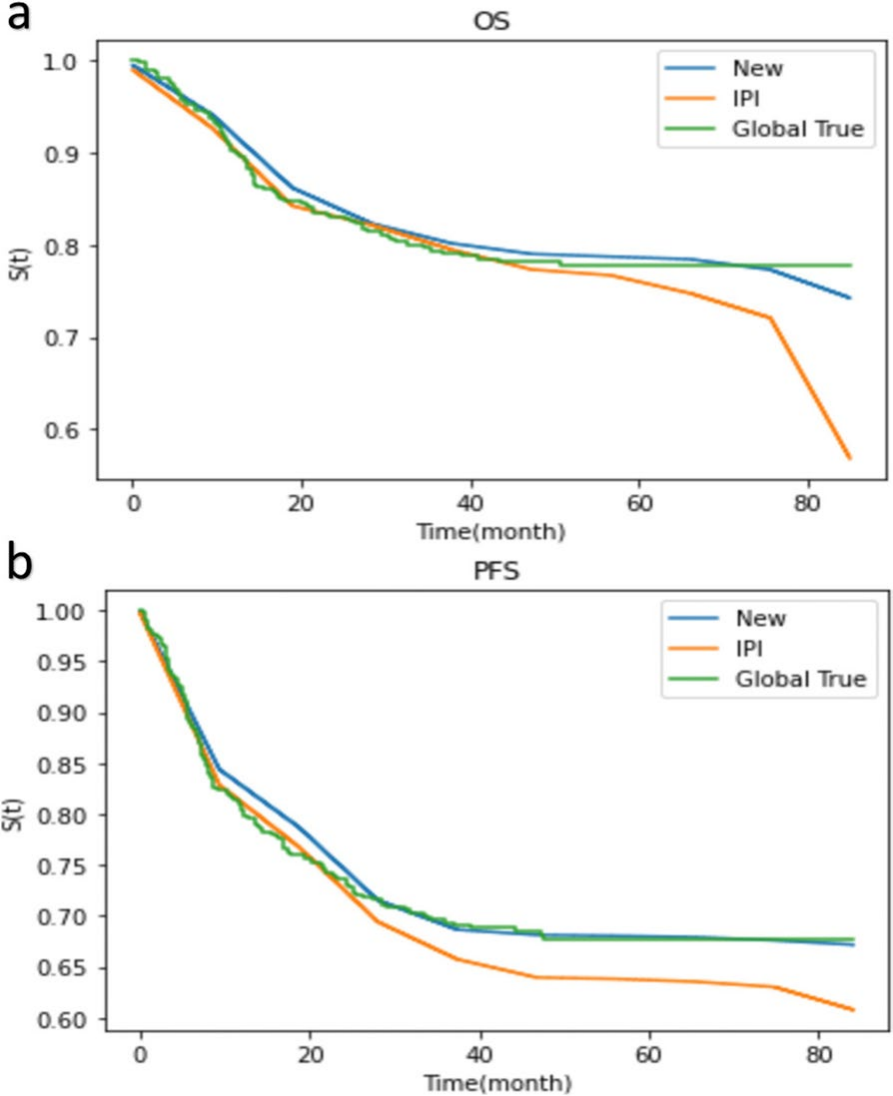
Model fit for OS (**a**) and PFS (**b**). The closer the fitted curve is to the true curve, the higher the degree of fit of the model. OS, overall survival; PFS, progression-free survival; IPI, International Prognostic Index

To better evaluate the performance differences between the two prediction models and real-world outcomes, we conducted comparisons (Fig. 8). In terms of OS, the alignment of the IPI with the actual probability (global true) was slightly stronger than that of the new model within the first 40 months. However, the difference between the IPI and the global true became significantly larger thereafter than that of the new model, with the disparity gradually increasing over time. In general, the new model exhibited better alignment with the actual outcomes than the IPI (Fig. 8a).

In terms of PFS, both the new model and the IPI aligned well with the actual probability within the first 12 months (Fig. 8b). We found slight deviation between the new model’s predictions and the actual PFS between 12 and 30 months, and the new model’s predictions largely aligned with the global true after 30 months. By contrast, the deviation between the IPI and the actual PFS increased gradually over time. Overall, the predictive advantage of the new model for PFS was significant.

### Clinical application of the new sMcPM

The calculations required to operate the McPM are too complex to be widely applicable in clinical practice. Therefore, we designed a simplified version, the sMcPM. According to the weights of the variables in Table 2, we selected the top five contributors to patient survival (extranodal involvement sites, LDH, *MYC* gene status, AMC, and PLT). We integrated the five variables considering their relative weights; Table 5 shows the scoring criteria. Our sMcPM classified patients into three risk subgroups: a score of 0–2 indicates a low risk of poor outcomes, a score of 3–4 indicates intermediate risk, and a score of 5–7 indicates high risk.

**Table 5:** Comparison of sMcPM and IPI for stratifying patients into risk groups according to survival outcomes

**Model**	**Point allocation for prognostic factors**	**n (%)**	**OS**			**PFS**		
			**1-year OS**	**3-year OS**	**5-year OS**	**1-year PFS**	**3-year PFS**	**5-year PFS**
**New score (sMcPM)**								
Low risk (0-2)	Extranodal sites: 0 or 1=0	125 (31.2%）	91.70%	84.00%	82.70%	82.70%	74.70%	73.80%
Intermediate risk (3-4)	Extranodal sites: 2 or 3=1	239 (59.6%)	86.90%	67.60%	65.60%	73.90%	56.80%	52.50%
High risk (5-7)	Extranodal sites: > 3=2	37 (9.2%)	63.30%	46.20%	46.20%	58.80%	30.80%	30.80%
	LDH: > 240 (U/L)=1							
	AMC: > 0.6* (10^9/L)=1							
	PLT: < 100* (10^9/L)=1							
	MYC gene status: positive=2							
**IPI**								
Low risk (0-1)	Extranodal sites: > 1=1	190 (47.4%)	96.30%	88.90%	86.80%	91.10%	83.70%	80.30%
Low‐intermediate risk (2)	LDH: > ULN=1							
	Age: > 60=1	86 (21.4%)	88.40%	79.10%	79.10%	71.20%	61.60%	61.60%
Intermediate‐high risk (3)	ECOG: > 1=1							
	Stage: III or IV=1	75 (18.7%)	84.50%	68.70%	68.00%	73.30%	54.70%	54.70%
High risk (4-5)		50 (12.5%)	72.70%	58.00%	55.40%	63.00%	50.00%	50.00%

*Abbreviations*: *IPI* International Prognostic Index, *sMcPM* simplified McPM, *OS* overall survival, *PFS* progression-free survival, *LDH* lactate dehydrogenase, *AMC* absolute monocyte count, *PLT* platelet count, *ECOG* Eastern Cooperative Oncology Group

**Fig. 9:**
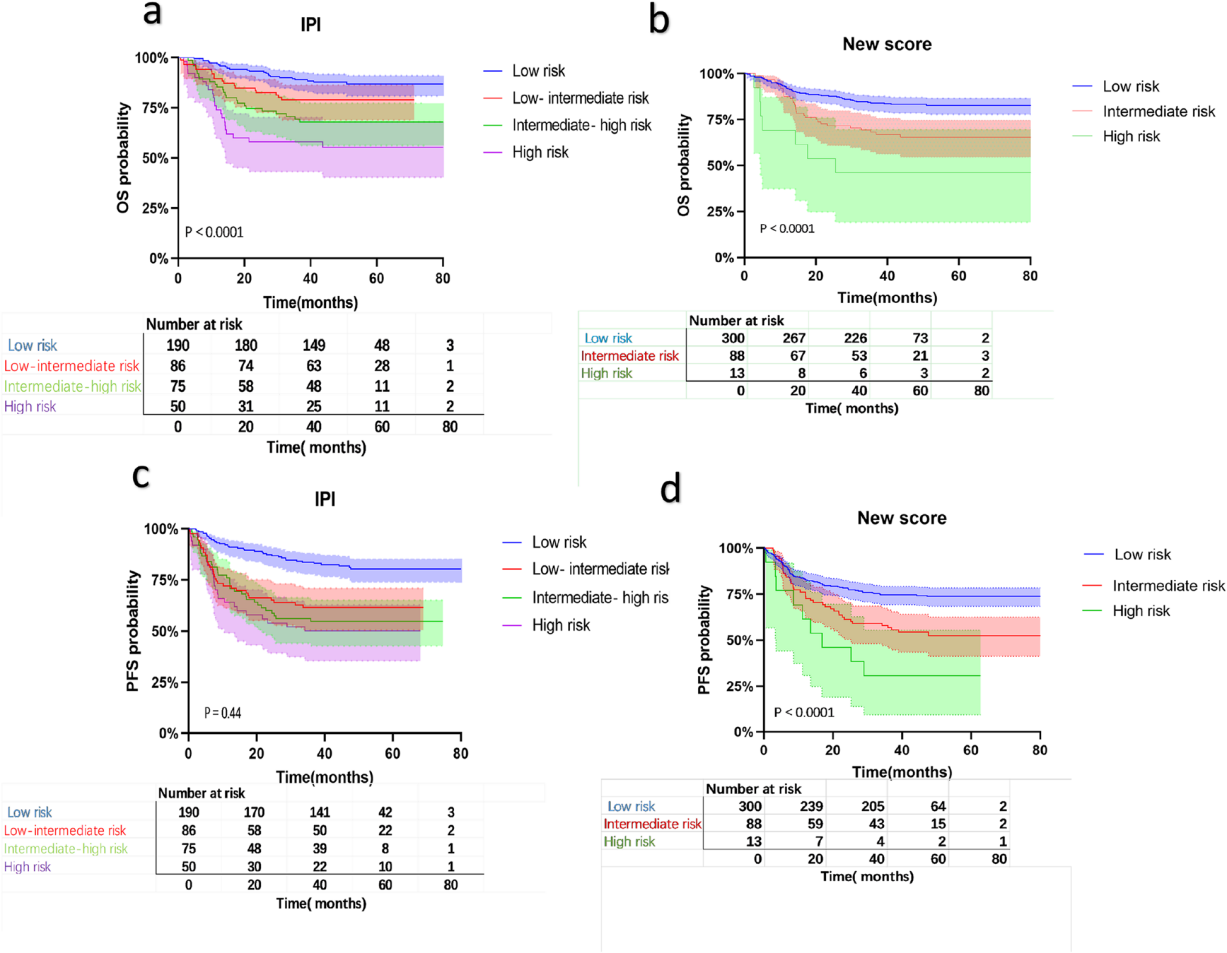
Performance of the IPI (**a**) and the new score (sMcPM) (**b**) in terms of stratifying patients according to OS and PFS [(**c**) and (**d**), respectively]. IPI and new score are similar in prognostic stratification of OS, but new score has an advantage in PFS. OS, overall survival; PFS, progression-free survival; IPI, International Prognostic Index

 We further compared the OS and PFS of the different risk groups according to the two models (Fig. 9) to visualize the stratification by prognosis. The stratification performance of OS based on the IPI and sMcPM was similar and significant in both cases (both *P* < 0.0001). However, stratification by IPI of the PFS was inferior to that of the sMcPM, and the former could not distinguish intermediate- from high-risk patients (*P* = 0.44 vs. *P* < 0.0001). The 1-year PFS rates of the four IPI groups were 91.10%, 71.20%, 73.30%, and 63.00% (Table 5). The 1‐year PFS rates of the three sMcPM groups (low-, intermediate-, and high-risk groups) were 82.70%, 73.90%, and 58.8%. Thus, sMcPM was superior at identifying high-risk patients, stratifying patients, and predicting their prognoses. More detailly, the COX for OS and PFS for IPI, sMcPM, and both variables together is included in Table S3. In conclusion, the new simplified model fully considers the important influence of molecular factors, transforms numerical variables into binary variables and constitutes a more stable, reliable, and applicable stratification tool for patients with DLBCL. These results suggest that our sMcPM could serve as a reliable prognostic tool.

## Discussion

DLBCL is the most common subtype of NHL, accounting for approximately 30–40% of all cases [32]. More than 50% of patients are cured with the first-line RCHOP regimen, but a small proportion still have a poor prognosis [33, 34]; identifying these high-risk patients is important. IPI, R-IPI [35], and NCCN-IPI [36] are widely used indexes for prognostic assessment, but none of them can clearly identify individuals with a long-term survival < 50% during rituximab treatment [16, 37]. In 2016, given the prognostic importance of *MYC*, *BCL2*, and *BCL6* rearrangements in patients with lymphoma [34, 38–40], the World Health Organization (WHO) defined a new entity with an aggressive clinical course and poor clinical outcome, high-grade B-cell lymphomas with *MYC* and *BCL2* and/or *BCL6* rearrangements (HGBL-R), which was previously known as DHL/THL [41–44]. In other words, molecular heterogeneity is an important factor in the prognosis of patients with DLBCL, but existing scoring systems do not meet the demands of the molecular era. There are two main limitations of the IPI model. First, it only includes clinical indicators and ignores the important impact of molecular genetic heterogeneity on prognosis. Second, the use of Cox survival analysis alone to screen for independent prognostic factors is relatively outdated and cannot account for the non-linear associations between complex and diverse variables [45, 46]. Relevant studies showed that even with the same IPI score, the prognoses of patients with DLBCL may differ significantly [5, 17]. Thus, we created the McPM for patients with DLBCL to integrate the complex prognostic factors in the molecular era.

The incorporation of prognostic indicators linked to molecular genetics is one of the two main strengths of our McPM. Domestic and international studies have confirmed the prognostic significance of *MYC*, *BCL2*, and *BCL6* gene abnormalities in patients with DLBCL [17, 19, 45]. Tzankov et al. analysed 432 patients and found that those with *MYC* gene rearrangements had a worse prognosis than those without (median OS rate, 42% vs. 86%, *P* = 0.038; median PFS, 42 vs. 75 months, *P* = 0.049) [47]. A similar study reported that patients with *MYC* amplification also had poorer OS than those with normal *MYC* gene status, as did those with rearrangements (both *P* < 0.01) [48]. Obermann et al. performed FISH testing of *BCL2* gene status in 224 patients with newly diagnosed DLBCL. In patients with the non-GCB subtype, the presence of any *BCL2* gene abnormality correlated with a shorter median OS (12 vs. 109 months, *P* = 0.003) [49]. Similarly, Huang et al. showed that the prognosis of patients with *BCL2* gene rearrangement or amplification was significantly worse than that of patients with normal gene status [50]. Akyurek et al. analysed the gene rearrangement status of 239 DLBCL cases and found that patients with *BCL6* rearrangements, particularly those with the non-GCB subtype, had a worse prognosis, suggesting that *BCL6* rearrangement may be a biomarker of an aggressive disease course in non-GCB subgroups [17]. In another study, a trend toward inferior OS was observed in patients with *BCL6* rearrangements who received immunochemotherapy (*P* = 0.08) [51]. It is worth noting that we previously found that patients with *MYC* and *BCL2* and/or *BCL6* gene rearrangements exhibited an aggressive clinical course and a poor response to a first-line RCHOP regimen, with 5-year OS and PFS rates of 27% and 18%, respectively [52]. In conclusion, *MYC*, *BCL2*, and *BCL6* are associated with poor prognosis, and their inclusion in our scoring system facilitates stratification.

The screening method for prognostic indicators is another advantage of our McPM. We used artificial intelligence (AI) to create a new prognostic model with potential to aid accurate diagnosis, treatment, and prognostic assessment of tumours [53]. After collecting 22 variables, including clinical and pathological features and laboratory and molecular genetic test results, from 401 patients with DLBCL in our center, we used the RSF method to demonstrate the importance of each variable. We obtained identical results using the VIMP and minimum depth methods in terms of the variables selected. A large VIMP means that a variable has a large impact on model accuracy and is therefore important. By contrast, the minimum depth method assigns smaller values to more important variables. The selection of the same variables by the two methods confirmed the prognostic value thereof. We constructed the McPM based on Bi-LSTM and logistic hazard techniques. Similar models used for other cancers, including lung cancer [54] and nasopharyngeal cancer [55], achieved early successes. We believe that our AI-based approach can deal with complex non-linear associations between variables and has unique advantages over traditional Cox regression analyses when dealing with survival data [27].

We compared the OS and PFS predictions between the IPI and McPM in several respects. Compared with the IPI, the McPM had a higher C-index and a lower BS for OS and PFS. According to the 1-, 3-, and 5-year ROC curve analyses for OS, the area under the curve of the McPM was larger than that of the IPI. Notably, our results supported an association between survival time and the AUC value. With longer survival times, the gap between the McPM and IPI results gradually widened; the AUC value of the new model was consistently high, while that of the IPI decreased gradually. This implies that the McPM has a clear advantage for predicting long-term survival. The McPM can identify a high-risk subgroup of patients with long-term survival < 50%, which many other models have failed to achieve [16]. In the comparison of the ROC curves for PFS, the area under the curve of the McPM was similar to or larger than that of the IPI, which suggests that the McPM is helpful to identify patients with poor treatment responses and predict disease progression, which are important factors for appropriate, individualized treatment. According to the AUC values (Fig. 5b) for PFS, the two models in this study differed within and after 1 year of diagnosis, which may further reflect the heterogeneity of DLBCL. Future clinical assessment could be improved by combining the two scoring systems. By comparing the predictions and actual outcomes, we found that the results of the McPM were more accurate than those of the IPI. In conclusion, the McPM had better prediction accuracy and stability, achieved by the integration of comprehensive prognostic indicators. The McPM could predict individual events, and we believe it is a suitable stratification tool for the molecular era.

The McPM calculation process is too complicated to be used directly in the clinical practice. Therefore, we established a simplified model, the sMcPM, by selecting 5 from among the 22 variables according to their weights (extranodal involvement sites, LDH, MYC gene status, AMC, and PLT). The number of extranodal involvement sites was the most important factor in our study. Moreover, we explored the impact of different numbers of extranodal sites on patient survival. Patients with more sites of extranodal involvement seemed to have a worse prognosis, similar to the findings of El-Galaly et al. [56]. LDH, a meaningful tumour biomarker, is often negatively correlated with prognosis [57]. A relevant study suggested that LDH may reflect the severity of disease [58]. *MYC* gene status is the only molecular genetic factor included in our scoring system. Studies have shown that *MYC* mutations are often associated with a poor prognosis [48, 52, 59]. Monocyte counts are also associated with the survival and prognosis of patients with lymphoma [60, 61]. Platelets play an important role in tumour immunity and angiogenesis; both factors are closely related to prognosis [62–64]. Our new model comprises these five indicators and is easy to use for clinicians. The sMcPM performs similarly to the IPI in terms of predicting OS, but it has a significant advantage in PFS prognostic stratification. Studies have shown that neither the IPI nor the R-IPI can define a high-risk group with a 3-year PFS < 50% [56], while the sMcPM can identify a group of patients with a 3-year PFS of 30.8%. This suggests that the indicators included in our new model have an important impact on disease progression, and our model may help guide subsequent clinical treatment with new drugs. We believe the sMcPM may become a widely used stratification tool in the molecular age.

FISH is not a routine test in clinical practice due to its high costs [34]. Therefore, few studies with large samples have been able to show complete gene rearrangements in patients with DLBCL, which limits the assessment of their prognostic impact. In response to this situation, we incorporated 22 variables from 401 patients, including both clinical and molecular factors, to construct a prognostic scoring system considering molecular heterogeneity. While the establishment of the McPM is a step forward, there are some limitations. The McPM includes too many factors, which makes it difficult to calculate and is therefore not suitable for being used in clinical practice. Important information is retained in the simplified sMcPM. However, the new score did not outperform the IPI in terms of OS prediction. In addition, as this is a retrospective study, validation in a large cohort is needed.

## Conclusions

We established a new prognostic model that is superior to the IPI in terms of prognostic prediction accuracy and stability. Moreover, the sMcPM can better meet the demand for prognostic stratification in the molecular era, and we expect it will become a widely used stratification tool with the ability to guide personalized clinical treatments.

### Supplementary Information

Supplementary Material 1.

Supplementary Material 2.

Supplementary Material 3.

## Data Availability

The dataset used and analyzed during the current study are available from the corresponding author on reasonable request.

## Abbreviations

NHL: Non-Hodgkin lymphoma
DLBCL: Diffuse large B-cell lymphoma
RCHOP: Rituximab plus cyclophosphamide, doxorubicin, vincristine and prednisone
IPI: International Prognostic Index
R-IPI: Revised International Prognostic Index
NCCN-IPI: National Comprehensive Cancer Network IPI
TAMs: Tumour-associated macrophages
*MYC-R*: *MYC* Rearrangement
*BCL2-R*: *BCL2* Rearrangement
*BCL6-R*: *BCL6* Rearrangement
SHL: Single-hit lymphoma
FISH: Fluorescence situ Hybridization
DAPI: 4',6-Diamidino-2-phenylindole
HE: Hematoxylin-eosin
OS: Overall survival
PFS: Progression free survival
VIMP: Variable importance
RSF: Random Survival Forest
McPM: Molecular-contained Prognostic Model
Bi-LSTM: Bidirectional long-short term memory
AMC: Absolute monocyte count
PLT: Platelet
LDH: Lactate dehydrogenase
ECOG: Eastern Cooperative Oncology Group
C-index: Concordance index
BS: Brier Score
DHL/THL: Double-hit/Triple-hit lymphoma
IBS: Integrated Brier Score
ROC: Receiver operating characteristic
AUC: Area under curve
sMcPM: Simplified McPM
WHO: World Health Organization
(HGBL,R): High Grade B-cell Lymphomas with MYC and BCL2 and/or BCL6 rearrangements
AI: Artificial Intelligence
