# Supplementary material for: Artificial intelligence-based prognostic model accurately predicts the survival of patients with diffuse large B-cell lymphomas: analysis of a large cohort in China
Source: BMC Cancer. 2024 May 22;24:621. doi: 10.1186/s12885-024-12337-z (PMC11110380; doi:10.1186/s12885-024-12337-z)
Supplement: Supplementary file 1 — Supplementary Material 1. [file 12885_2024_12337_MOESM1_ESM.docx]

**Table S1 Univariate of Table1 variables**

| Variables | No. | PFS | OS |  |
| --- | --- | --- | --- | --- |
|  |  | p-value | p-value |  |
| Age, >60 vs. ≤60 (year) | 170 vs. 231 | 0.297 | 0.032* |  |
| Gender, female vs. male | 191 vs. 210 | 0.037* | 0.088 |  |
| Ann Arbor stage, III–IV vs. I–II | 210 vs. 191 | ＜0.001* | ＜0.001* |  |
| ECOG, ≥2 vs.＜2 | 60 vs. 341 | ＜0.001* | 0.003* |  |
| B symptoms, with vs. without | 85 vs. 316 | 0.938 | 0.884 |  |
| Extranodal sites, ≥2 vs.＜2 | 93 vs. 308 | ＜0.001* | ＜0.001* |  |
| ALC, ≤1.0 vs.＞1.0 (×10^9/L) | 114 vs. 287 | 0.028* | 0.002* |  |
| AMC, ≥0.6 vs.＜0.6 (×10^9/L) | 136 vs. 265 | 0.003* | ＜0.001* |  |
| ALC/AMC: ＜3:1 vs. ≥ 3:1 | 197 vs. 204 | 0.092 | 0.004* |  |
| Albumin, ＜35 vs. ≥ 35 (g/L) | 41 vs. 360 | 0.801 | 0.224 |  |
| β2 microglobulin, ＞3.0 vs. ≤3.0 (mg/L) | 97 vs. 304 | ＜0.001* | ＜0.001* |  |
| LDH, ＞240 vs. ≤240 (U/L) | 194 vs. 207 | ＜0.001* | ＜0.001* |  |
| IPI, 0–2 vs. 3-5 | 276 vs. 125 | ＜0.001* | ＜0.001* |  |
| Ki-67, ＞70% vs. ≤70% | 238 vs. 163 | 0.712 | 0.886 |  |
| COO, Non-GCB vs. GCB | 274 vs. 127 | 0.739 | 0.437 |  |
| *MYC* gene, rearrangement vs. amplification vs. normal | 40 vs. 75 vs. 286 | 0.003* | ＜0.001* |  |
|  |  |  |  |  |
| *BCL2* gene, rearrangement vs. amplification vs. normal | 21 vs. 137 vs. 243 | 0.003* | 0.014* |  |
|  |  |  |  |  |
| *BCL6* gene, rearrangement vs. amplification vs. normal | 145 vs. 104 vs. 152 | 0.325 | 0.704 |  |
|  |  |  |  |  |
| Double-/triple-hit, yes vs. no | 16 vs. 385 | 0.011* | 0.022* |  |

*P<0.05 stands for statistical significance.

**Abbreviations: PFS, progression-free survival; OS, overall survival; ECOG, Eastern Cooperative Oncology Group; ALC, absolute lymphocyte count; AMC, absolute monocyte count; LDH, lactate dehydrogenase; IPI, International Prognostic Index; COO, cell of origin; GCB, germinal-center B-cell**
