# Supplementary material for: Artificial intelligence-based prognostic model accurately predicts the survival of patients with diffuse large B-cell lymphomas: analysis of a large cohort in China
Source: BMC Cancer. 2024 May 22;24:621. doi: 10.1186/s12885-024-12337-z (PMC11110380; doi:10.1186/s12885-024-12337-z)
Supplement: Supplementary file 2 — Supplementary Material 2. [file 12885_2024_12337_MOESM2_ESM.docx]

**Table S2 Multivariate of table1 variables**

| Variables | PFS | | OS | |
| --- | --- | --- | --- | --- |
|  | OR (95%CI) | p-value | OR (95%CI) | p-value |
| Age, >60 vs. ≤60 (year) |  |  | 0.747（0.461-1.208） | 0.234 |
| Gender, female vs. male | 0.755(0.523-1.091) | 0.134 |  |  |
| Ann Arbor stage, III–IV vs. I–II | 0.528(0.327-0.853) | 0.009* | 0.656（0.355-1.214） | 0.18 |
| ECOG, ≥2 vs.＜2 | 0.865(0.523-1.430) | 0.572 | 1.034（0.581-1.838） | 0.911 |
| B symptoms, with vs. without |  |  |  |  |
| Extranodal sites, ≥2 vs.＜2 | 0.754(0.444-1.281) | 0.297 | 0.685（0.367-1.277） | 0.234 |
| ALC, ≤1.0 vs.＞1.0 (×10^9/L) | 0.905(0.610-1.344) | 0.621 | 0.707（0.432-1.159） | 0.169 |
| AMC, ≥0.6 vs.＜0.6 (×10^9/L) | 0.761(0.520-1.115) | 0.161 | 0.603（0.356-1.020） | 0.059 |
| ALC/AMC: ＜3:1 vs. ≥ 3:1 |  |  | 0.867（0.482-1.562） | 0.635 |
| Albumin, ＜35 vs. ≥ 35 (g/L) |  |  |  |  |
| β2 microglobulin, ＞3.0 vs. ≤3.0 (mg/L) | 0.854(0.570-1.280) | 0.445 | 0.75（0.463-1.213） | 0.240 |
| LDH, ＞240 vs. ≤240 (U/L) | 0.473(0.306-0.730) | 0.001* | 0.646（0.372-1.122） | 0.121 |
| IPI, 0–2 vs. 3-5 | 0.658(0.341-1.268) | 0.211 | 0.878（0.382-2.021） | 0.760 |
| Ki-67, ＞70% vs. ≤70% |  |  |  |  |
| COO, Non-GCB vs. GCB |  |  |  |  |
| *MYC* gene, rearrangement vs. normal | 1.803(0.973-3.343) | 0.061 | 2.949（1.483-5.861） | 0.002* |
| *MYC* gene, amplification vs. normal | 0.775(0.468-1.284) | 0.323 | 0.959（0.518-1.775） | 0.894 |
| *BCL2* gene, rearrangement vs. normal | 1.149(0.500-2.642) | 0.744 | 1.622（0.651-4.044） | 0.299 |
| *BCL2* gene, amplification vs. normal | 2.004(1.341-2.993) | 0.001* | 1.824（1.122-2.968） | 0.015* |
| Double-/triple-hit, yes vs. no | 0.893(0.346-2.304) | 0.815 | 1.355（0.452-4.064） | 0.588 |

*P<0.05 stands for statistical significance.

**Abbreviations: PFS, progression-free survival; OS, overall survival; ECOG, Eastern Cooperative Oncology Group; ALC, absolute lymphocyte count; AMC, absolute monocyte count; LDH, lactate dehydrogenase; IPI, International Prognostic Index; COO, cell of origin; GCB, germinal-center B-cell**
