# Supplementary material for: Artificial intelligence-based prognostic model accurately predicts the survival of patients with diffuse large B-cell lymphomas: analysis of a large cohort in China
Source: BMC Cancer. 2024 May 22;24:621. doi: 10.1186/s12885-024-12337-z (PMC11110380; doi:10.1186/s12885-024-12337-z)
Supplement: Supplementary file 3 — Supplementary Material 3. [file 12885_2024_12337_MOESM3_ESM.docx]

**Table S3 COX for OS and PFS for IPI, sMcPM, and both variables**

| Outcome | Variables | No. | Univariate | Multivariate |
| --- | --- | --- | --- | --- |
|  |  |  | p-value | p-value |
| OS | Age, >60 vs. ≤60 (year) | 170 vs. 231 | 0.33 |  |
|  | Ann Arbor stage, III–IV vs. I–II | 210 vs. 191 | ＜0.001* | 0.268 |
|  | LDH, ＞UNL vs. ≤ULN | 194 vs. 207 | ＜0.001* | 0.232 |
|  | ECOG, ≥2 vs.＜2 | 60 vs. 341 | 0.004* | 0.813 |
|  | Extranodal sites, ≥2 vs.＜2 | 93 vs. 308 | ＜0.001* | 0.391 |
|  | AMC, ≥0.6 vs.＜0.6 (×10^9/L) | 136 vs. 265 | ＜0.001* | 0.016* |
|  | PLT, ＜100 vs. ≥100 (×10^9/L) | 11 vs. 390 | 0.014* | 0.116 |
|  | *MYC* gene, positive vs. normal | 115 vs. 286 | 0.010* | 0.041* |
|  | IPI, 0-1 vs. 2 vs. 3 vs. 4-5 | 190 vs. 86 vs. 75 vs. 50 | ＜0.001* | 0.903 |
|  | sMcPM, 0-2 vs. 3-4 vs. 5-7 | 125 vs. 239 vs. 37 | ＜0.001* | 0.790 |
|  |  |  |  |  |
| PFS | Age, >60 vs. ≤60 (year) | 170 vs. 231 | 0.298 |  |
|  | Ann Arbor stage, III–IV vs. I–II | 210 vs. 191 | ＜0.001* | 0.049* |
|  | LDH, ＞UNL vs. ≤ULN | 194 vs. 207 | ＜0.001* | 0.006* |
|  | ECOG, ≥2 vs.＜2 | 60 vs. 341 | ＜0.001* | 0.155 |
|  | Extranodal sites, ≥2 vs.＜2 | 93 vs. 308 | ＜0.001* | 0.127 |
|  | AMC, ≥0.6 vs.＜0.6 (×10^9/L) | 136 vs. 265 | 0.004* | 0.103 |
|  | PLT, ＜100 vs. ≥100 (×10^9/L) | 11 vs. 390 | 0.132 |  |
|  | *MYC* gene, positive vs. normal | 115 vs. 286 | 0.040* | 0.194 |
|  | IPI, 0-1 vs. 2 vs. 3 vs. 4-5 | 190 vs. 86 vs. 75 vs. 50 | ＜0.001* | 0.372 |
|  | sMcPM, 0-2 vs. 3-4 vs. 5-7 | 125 vs. 239 vs. 37 | ＜0.001* | 0.915 |

*P<0.05 stands for statistical significance.

**Abbreviations: LDH, lactate dehydrogenase; ECOG, Eastern Cooperative Oncology Group; AMC, absolute monocyte count; PLT, platelet count; IPI, International Prognostic Index; sMcPM, simplified McPM; OS, overall survival; PFS, progression-free survival**
